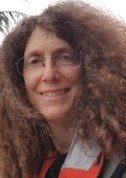# Beyond Charge Balance: Counter‐Cations in Polyoxometalate Chemistry

**DOI:** 10.1002/anie.201905600

**Published:** 2019-10-31

**Authors:** Archismita Misra, Karoly Kozma, Carsten Streb, May Nyman

**Affiliations:** ^1^ Institute of Inorganic Chemistry I Ulm University Albert-Einstein-Allee 11 89081 Ulm Germany; ^2^ Department of Chemistry Oregon State University Corvallis OR 97331 USA

**Keywords:** Cations, Composites, Interactions, Polyoxometalates, Supramolecular Chemistry

## Abstract

Polyoxometalates (POMs) are molecular metal‐oxide anions applied in energy conversion and storage, manipulation of biomolecules, catalysis, as well as materials design and assembly. Although often overlooked, the interplay of intrinsically anionic POMs with organic and inorganic cations is crucial to control POM self‐assembly, stabilization, solubility, and function. Beyond simple alkali metals and ammonium, chemically diverse cations including dendrimers, polyvalent metals, metal complexes, amphiphiles, and alkaloids allow tailoring properties for known applications, and those yet to be discovered. This review provides an overview of fundamental POM–cation interactions in solution, the resulting solid‐state compounds, and behavior and properties that emerge from these POM–cation interactions. We will explore how application‐inspired research has exploited cation‐controlled design to discover new POM materials, which in turn has led to the quest for fundamental understanding of POM–cation interactions.

## Graphical abstract

**Supporting and lead roles**: Beyond the classic roles of counter‐cations for polyoxometalates (POMs), there is emerging and compounding evidence that they drive reactivity; template cluster growth and supramolecular assembly; and endow POMs and POM materials with function. This Review covers both the classic and emerging roles of counter‐cations in fundamental and applied POM science.

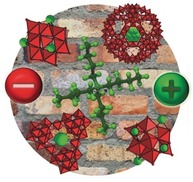

## 1 Introduction: Fundamentals of POM–Cation Interactions

### 1.1 Polyoxometalates

Polyoxometalates (POMs) are molecular metal‐oxide anions that form by self‐assembly of reactive oxometalate precursors in aqueous or organic solution.^1^ They are key intermediates in the reaction pathway from water‐soluble metal ions to insoluble metal oxides, and isolation of these enable elucidation and control over reaction pathways. POMs are structurally and chemically diverse with reactivities leading to use in catalysis^2^ and sustainable energy,^3^ electronics,^4^ sensors,^5^ radionuclide capture^6^, ^7^ and biomedical applications.^8^ POMs are most traditionally composed of high‐valent (typically d^0^ or d^1^) group V and VI transition metals (V, Mo, W, Nb, and Ta). Counter‐cations are imperative to isolate pure‐phase POMs; but their role goes far beyond simple charge‐balance.

In this Review, we explore the importance of cation–POM interactions and describe how these interactions can direct structure and reactivity of POMs across multiple length scales from the molecular through the device level. The detailed analysis of our current understanding of POMs and counter‐cations will shed light on emerging research areas that impact the broader scientific community. We will explore the role of cations on individual POM molecules, POM aggregates and POM crystals to showcase how cation and POM structure can be combined to yield new fundamental and applied chemistry. The aim of this Review is to demonstrate that cations are not simple spectator ions but are key players in 21st century POM science. In the following sections, we briefly summarize the traditional roles of counter‐ions (i.e. solubility and templating effects), then provide a short overview of experimental and theoretical methods that rationalize POM–cation interactions. Then, we will explore the role of inorganic and organic cations on POM assembly and aggregation into complex superstructures. We will highlight how new materials and new function arise from suitable POM–cation combinations. Finally, we discuss emerging areas of POM–cation research, highlight future opportunities to glean fundamental insight, and describe new applications based on the synergistic roles of POMs and their counter‐cations.

### 1.2 POM–Cation Interactions

Electrostatics is the dominant POM–cation interaction, particularly when both species are in close contact, for example, in the solid state or in weakly coordinating solvents. While this describes classic POM cations (e.g. alkali/ alkaline earth cations); more complex cations exhibit divergent interaction modes including hydrogen‐bonding, ion–dipole, partially covalent and van‐der‐Waals interactions. Additionally, recent studies report cation–π‐interactions as a structure‐directing feature for POMs covalently functionalized with aromatic groups.^9^ Note that in many cases described here, complex combinations of these interactions can coexist, and assigning specific effects to one type of interaction is difficult. In the following sections, we will discuss these various POM–cation interactions.

### 1.3 Solubility and Ion‐Pairing

One of the most fundamental roles of cations in POM chemistry is controlling solubility. While many studies rely on solubility in various solvents, other applications require insolubility, for example, to prevent leaching from a material or device. Thus, understanding solubility trends is a key aspect of POM chemistry and is used in crystallization, purification, or colloid stabilization. Aqueous solubility trends were notably described for proteins by Hofmeister and co‐workers,^10^ and have also been extensively studied for polymers.^11^ Hofmeister's study investigated the critical concentration of counter‐ions to precipitate a protein. The solubility trend for cation–protein referred to as the Hofmeister series is NH_4_^+^ > Na^+^ > Li^+^ > Mg^2+^ > Ca^2+^ > guanidinium. The ability of these cations to precipitate proteins is related to their varying charge‐density which promotes ordering in water, leading to an increased hydrophobic effect that triggers protein aggregation and precipitation.^12^, ^13^, ^14^, ^15^

Considering the effects of POMs on the solution stability of macromolecules, we note that POMs feature a large hydration shell, show high polarizability and low charge‐density.^16^, ^17^ Consider the prototype Keggin anion [SiW_12_0_40_]^4−^: while the total charge is relatively high (4−), the charge density is rather low (i.e. charge divided by number of atoms: −4/53=−0.075). This is compared with oxoanions such as sulfate, where the charge‐density is −2/5 (=−0.4) or chloride, where the charge‐density is −1. In addition, when comparing the charge density trends of different POM classes, the charge‐to‐metal ratio *q*/*M* has recently been introduced as an important reactivity criterion.^18^, ^19^ For example, W^VI^‐based tungstates feature low *q*/*M* ratios (e.g. [PW_12_O_40_]^3−^, *q*/*M*=0.25; [P_2_W_18_O_62_]^6−^, *q*/M=0.33, while the Nb^V^ species [Nb_6_O_19_]^8−^ feature *q*/*M*=1.33.

In sum, these properties render POMs chaotropic anions^20^ that disrupt hydrogen bonding in aqueous solvents,^16^, ^21^, ^22^ so that POMs promote the precipitation of macromolecules such as polymers, neutral surfactants, and proteins from water. Given the broad solvent range under which POMs are employed, careful consideration of the solvation energies of POM anion and corresponding cation is required, as the interplay between solvation energies and lattice energies of the respective POM–cation salt essentially control the solubility of the species under investigation.

**Figure 1:**
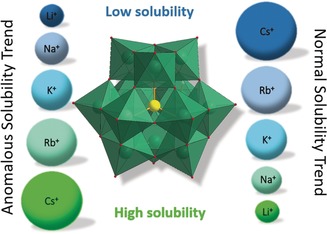
General solubility trends observed for POM anions (the Keggin ion [XM_12_O_40_]^*n*−^ (X often P, Si, M=Mo, W) is used for illustrative purposes) salts with alkali metal cations. Normal solubility trend is observed for most POM salts including Mo, V, and W, while the anomalous trend is experienced mostly for Nb and Ta POMs. (Blue represents the least soluble, green the most soluble agents.).

Missing from the Hofmeister explanation or any solvent‐centric models describing solubility is the effect of cation–anion interactions, and specific structures of these interactions in solution. Intriguingly, solubility trends resulting from different POM–cation combinations differ significantly depending on the type of POM. Group 5/6 POMs that assemble in acid (V, Mo, and W) exhibit high aqueous solubility when paired with small alkali metal cations (Li^+^ and Na^+^), while their salts with larger alkali metal cations (e.g. Cs^+^) often show poor water‐solubility (Figure 1). This so‐called “normal” solubility trend is expected, since small cations (e.g. Li^+^) have a large, strongly bound hydration shell and cannot come in direct contact with the low charge‐density POMs, so precipitation initiated by ion‐pairing is deterred. In addition, few contacts and poor packing in the solid‐state lead to a low lattice stabilization energy, making precipitation energetically less favored. On the other hand, the large Cs^+^ ion readily binds electrostatically to POMs and forms insoluble aggregates—this behavior is exploited in POM heterogeneous catalysis.^23^ Solubility of these POMs in organic solvents (chlorinated, aromatic, polar or non‐polar) can be achieved by using large alkylammonium or alkylphosphonium cations.^24^, ^25^ In contrast to the solubility behavior of these classic POMs (formed under acidic conditions), POMs formed in base (Nb or Ta), exhibit higher solubility with larger alkali metal cations (e.g. Cs^+^), despite the fact they form contact ion‐pairs in solution.^26^ This surprising solubility behavior is dubbed “anomalous” or inverse solubility.^27^, ^28^ It is tempting to correlate this behavior with the pH‐stability of the POMs; however, note that the alkaline‐stable uranyl peroxide POMs exhibit normal solubility behavior.^7^, ^29^ The Nb/Ta POMs are also highly water‐soluble as tetramethylammonium (TMA) salts.^30^

It is more difficult to delineate solubility and ion‐pairing trends within a group (i.e. Nb compared to Ta, Mo compared to W) because isostructural analogues are few. However, Pfitzner and colleagues^16^ note stronger adsorption of the phosphotungstate Keggin ion to a nonionic micelle compared to the analogous phosphomolybdate Keggin, owing to lower charge density. Nyman and co‐workers observed different modes of ion‐pairing between Nb and Ta Lindqvist ions, and noted covalent character of the Cs−O bond in a Cs‐Lindqvist ion‐pair.^26^, ^31^

While still lacking an explanation, both the normal and anomalous solubility trends can be summarized as: 1) Low charge‐density cations paired with high charge‐density POMs are soluble, and 2) High charge‐density cations paired with low charge‐density POMs are soluble. These demonstrated and somewhat predictable solubility trends can be utilized for POM manipulation including crystallization, rapid dissolution, or precipitation.

### 1.4 Role of Cations in POM Assembly

The pivotal role of cations in POM chemistry is exemplified in the electrostatic stabilization of reactive lacunary POMs. Pioneering studies on POM stability and reactivity focused on the Keggin anion as a prototype metal oxide cluster (Figure 1). The Keggin anion features twelve metal–oxo octahedra assembled around a tetrahedral oxo‐anion, giving the generic formula [(X^*n*+^)M_12_O_40_]^(8−*n*)−^ (M=Mo, W; X=Si, P, less frequently Al, Ga, Ge, B, etc).^32^, ^33^ These clusters assemble in acid; pH≈1–3.^34^ Increasing pH leads to partial hydrolysis of the cluster, and formation of lacunary derivatives where one or several metal–oxygen moieties have been removed from the cluster shell. These structures are more reactive than the original Keggin anion due to the presence of multiple labile terminal oxo ligands. Electrostatic binding of alkali metal counter cations (particularly Na^+^ and K^+^) to these oxo ligands is imperative to stabilize and isolate these reactive species. Based on this principle, a family of transition‐metal‐functionalized lacunary Keggin anions has been established with applications ranging from catalysis to electronics and magnetism, and metal ions ranging from main group to transition metals and f‐elements.^35^, ^36^, ^37^, ^38^, ^39^

### 1.5 Experimental and Theoretical Methods to Explore Cation–POM Assembly

Typically, single‐crystal X‐ray diffraction (and more rarely powder X‐ray diffraction) offers initial insights into POM–cation interactions in the solid state. This data can serve as a starting point to assess the more complex and dynamic interactions between POMs and their cations in solution. Ion association in solution can generally be described by the formation of contact‐ion pairs, solvent‐shared/solvent‐separated ion pairs and fully‐solvated ion pairs.^40^ To rationalize these interactions in solution, a number of experimental and theoretical techniques have been established.

One classical method to probe ion‐pairing in solution is conductivity measurements. Conductivity in solution decreases with increasing ion‐pairing, as the number of unpaired ions available for charge transport in solution decreases. Additionally, the increased diameter of paired ions leads to lower mobility in solution.^41^ In principle, this enables quantitative determination of ion‐pairing based on conductivity and impedance spectroscopy.^42^

X‐ray scattering yields direct molecular‐level information on solution‐phase ion‐pairing. Over the last decade, pioneering studies exploiting small‐angle X‐ray scattering (SAXS) have yielded information on POM speciation and POM–counterion interactions.^43^ The high scattering contrast between the solvent and POM/cation metals provides information on POM–cation aggregation based on the interpretation of size (via Guinier approximation), shape (via pair distance distribution function (PDDF)) and purity of species.^44^ More detailed information is provided by total X‐ray scattering, where pair distribution function (PDF) and radial distribution function (RDF) analyses yield structural information at the atomic scale by monitoring atom‐pair correlations in individual clusters.^45^ Using this approach, contact–ion pairing in the alkali metal‐Lindqvist (Nb/Ta) system was studied; and semi‐quantitative determination of the cation‐number associated with a POM anion is possible.^31^, ^46^ Neutron diffraction and scattering can provide complementary information since neutrons are more sensitive to the light elements and several specific isotopes, providing a full structural and topological study on an aqueous‐phase POM–counterion system.^47^, ^48^ Anomalous SAXS that exploits different X‐ray wavelengths to highlight scattering of different elements similarly enhances detailed understanding of solution‐phase ion‐pairing.^49^

Nuclear magnetic resonance (NMR) measurements can probe cation–POM interactions in solution if suitable NMR‐active cations are present. In a recent study, Nyman and colleagues examined ion‐pairing between Cs^+^ cations and Nb/Ta POM anions using inversion‐recovery ^133^Cs‐NMR spectroscopy and compared quadrupolar relaxation rates that change as a function of association to the POM.^31^, ^46^ Static and dynamic light scattering can provide insights into POM–cation aggregation when larger colloid particles (≫1 nm) are present. This principle has been used to detect POM colloid formation under catalytic conditions.^50^ Over the past decade, cryogenic transmission electron microscopy (cryo‐TEM) has become a key method to explore nanostructures in frozen solution. This allows direct imaging of POMs in solution and eliminates artifacts of the deposition and drying processes. Pioneering studies have provided visual evidence of POM–counterion interactions such as cation‐promoted POM dimerization^51^ and POM superstructure aggregation.^52^ To date, cryo‐TEM imaging of POMs is still challenging due to the complex sample preparation, electron‐beam damage, and poor resolution at the atomic level. However, we expect that cryo‐TEM will become more widely used for POM studies as instrumentation and software improves, and this rare capability becomes more common.

Finally, theoretical studies are valuable to model POM–cation interactions.^53^ On the molecular level, density functional theory (DFT) and time‐dependent DFT (TD‐DFT) can provide detailed information on POM–cation interactions, binding modes and resulting changes of geometry and electronic structure.^54^ Thereby, experimentally accessible data such as vibrational or electronic absorption properties can be calculated from first principles, and characteristic features of the POM–cation interactions can be identified. Information on dynamic processes of POM–cation interactions can be accessed by classical and ab initio molecular dynamics (MD) simulations.^55^, ^56^, ^57^, ^58^ For example, MD was used in conjunction with the above‐mentioned microscopy study, and both independently showed formation of POM‐pairs linked by Na^+^, also consistent with the solid‐state structure.^51^ In summary, the combination of experiment and theory can potentially provide powerful and accurate information about POM–cation interactions in solution, guiding future developments of new POM architectures and superstructures.

## 2 Inorganic Cation–POM Materials: Structure‐Induced Functions

The widespread use of metal counter‐cations in POM chemistry has enabled breakthroughs ranging from fundamentally new POM architectures to technology advances in energy conversion. In this section, we examine how cations can tune structure and function of POMs. In addition to architectural considerations, we will focus on new reactivity introduced by the cation and emphasize areas where further research is needed to rationalize the exact function of the cation.

### 2.1 Cation‐induced stabilization and functionalization of metal–oxo clusters

Since the pioneering structural studies of 12‐phosphotungstic acid by J. F. Keggin (a student of W. L. Bragg) in 1933,^59^ most POM research has been focused on clusters based on Group 5/6 transition metals (mainly V, Mo and W). Here, cluster growth termination is achieved by the formation of terminal M=O multiple bonds with low nucleophilicity (requiring high‐valent transition metals to form d–p π‐bonds). The assembly of metal–oxo clusters based on lower‐valent transition metals has become possible by using stabilizing terminal ligand anions such as oxoanions (e.g. PO_4_^3−^, AsO_4_^3−^) or alkoxides, giving rise to “non‐classical” POM‐related species based for example, on Ti,^60^ Mn,^61^ Cu,^62^ Au,^63^ Pd,^64^ or Pt.^65^, ^66^ Note that these species should be distinguished from classical POMs due to their different type of addenda metal, the metal oxidation states and the alternative growth‐termination mechanism. In the authors’ view, they form the bridge between polyoxometalate chemistry and polynuclear metal–oxo coordination chemistry.

**Figure 2:**
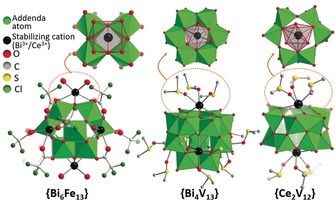
Structural representation of the cation‐stabilized POMs **{Bi_6_Fe_13_}**,^67^ **{Bi_4_V_13_},** 68 and **{Ce_2_V_12_}** described in Section 2.1; **{Ce_2_V_12_}**=[(Ce(dmso)_3_)_2_V^IV^V^V^_11_O_33_Cl]^2−^.^69^, ^70^

Recent pioneering studies have shown that multivalent metal cations can also cap growth and stabilize POMs and other anionic metal–oxo clusters. The following section will highlight recent examples of this approach with a focus on common stabilization routes and a summary of new compound classes accessed by this approach. In particular, the Bi^3+^ cation has been used to stabilize a range of new polyanion clusters. A ground‐breaking study by Nyman and colleagues described the isolation of an iron‐based Keggin anion by stabilization with Bi^3+^ cations. The species Bi_6_[FeO_4_Fe_12_O_12_(OH)_12_(O_2_CCCl_3_)_12_]^+^ (=**{Bi_6_Fe_13_}**) is obtained by reaction of Bi^3+^ and Fe^3+^ in water acidified with trichloroacetic acid.^67^ The compound is based on an all‐Fe^3+^ α‐Keggin framework with a 17− charge. The Bi^3+^ ions occupy tetragonal “vacancies” on the Keggin ion surface. Their coordination environment (CN=8, distorted square antiprism, *r*_ionic_ (Bi^3+^)_CN=8_=1.17 Å) is completed by four oxygen donor atoms from water or the trichloroacetate molecules that terminate the iron (Figure 2). This iron–oxo core is structurally related to ferrihydrite, prevalent in soil and biological iron storage systems such as ferritin.^67^ The authors determined that when Bi^3+^ is not present during synthesis, only colloidal ferrihydrite is formed. Further, when the Bi^3+^ cations are removed from **{Bi_6_Fe_13_}**, the molecular species rapidly convert to ferrihydrite colloids.

In an earlier study, Streb and colleagues used a similar approach to stabilize the elusive vanadium(V)‐based Keggin anion [V_13_O_40_]^15−^ with Bi^3+^ cations, also providing a molecular mimic of technologically important solid‐state bismuth vanadium oxides.^71^ The authors reported the self‐assembly of the first molecular Bi‐V‐oxide cluster H_3_[{Bi(dmso)_3_}_4_V_13_O_40_] (=**{Bi_4_V_13_}**) by reaction of Bi^3+^ with [H_3_V_10_O_28_]^3−^ in dimethyl sulfoxide (DMSO).^68^ The rare ϵ‐Keggin isomer^35^, ^72^ of **{Bi_4_V_13_}** is stabilized by four Bi^3+^ cations coordinated to the cluster surface (Figure 2), which in turn are coordinated by three terminal DMSO ligands. When considering the requirements to stabilize [ϵ‐V_13_O_40_]^15−^, it is apparent that structural/coordinative stabilization is required in addition to (cationic) electrostatic stabilization. In both **{Bi_6_Fe_13_}** and **{Bi_4_V_13_}**, there is a synergy between the Bi^3+^ stabilizing a highly charged polyanion cluster, and a coordinating ligand bound to the peripheral coordination site of Bi^3+^. This provides a conceptual strategy to further exploit Bi^3+^ for stabilizing highly charged clusters. Interestingly, Nyman and colleagues used this strategy in reverse, stabilizing a Bi–oxo cluster via encapsulation and weak coordination inside a uranyl peroxide fullerene‐like capsule.^73^

Further evidence for this multifunctional role of high‐valence metal cations includes the mixed‐valence Mo ϵ‐Keggin species [{La(H_2_O)_4_}_4_PMo^V^_8_Mo^VI^_4_O_36_(OH)_4_]^5+^ (**{La_4_Mo_12_}**).^74^ The species is structurally closely related to **{Bi_4_V_13_}**, featuring four La^3+^ cations, coordinated via three Mo−O−La bonds to the trigonal “vacancies” of the ϵ‐Keggin cluster. The authors show by ^31^P NMR spectroscopy that loss of one La^3+^ ion leads to destabilization and structural re‐arrangement of the ϵ‐Keggin geometry. Further studies of the complex showed that exchange of the La^3+^ with Ce^3+^ and other lanthanide cations is possible, thereby highlighting that cluster isolation by large cation stabilization could be of wide relevance for POM cluster development. This is also supported by recent reports which show that coordination of Ce^3+^ to vacant binding sites stabilizes novel polyoxovanadate clusters.^69^, ^70^ Finally, note that both **{Bi_6_Fe_13_}** and **{La_4_Mo_12_}** are metal–oxo cations—we will come back to this compound class in Section 4.

### 2.2 Cation‐Mediated POM Framework Assembly

Building on the principles described in Section 2.1, the design of framework materials where individual POMs are linked by metal‐based cations becomes possible. The emerging field of polyoxometalate open frameworks (POM‐OFs) has recently been reviewed^75^, ^76^ and in this section we will focus on porous frameworks formed by linking POM anions with metal cations. These systems hold great promise for application under chemically and thermally harsh conditions, however, the design and stabilization of systems with accessible pores—particularly when stable linkage in 3D is targeted—is still a major synthetic challenge.

**Figure 3:**
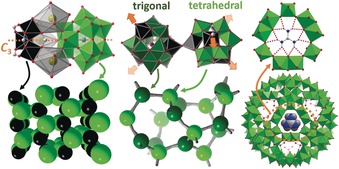
Structural representation of cation–POM frameworks described in Sections 2.2 and 2.3. Left: ionic zeotype frameworks reported by Mizuno, Uchida, and colleagues;^77^ center: Keggin‐net reported by Cronin and colleagues;^79^ right: guanidinium‐blocked **{Mo_132_}** Keplerate capsule reported by Müller and colleagues.^80^, ^81^

Mizuno, Uchida, and colleagues have established a simple and effective ionic assembly route leading to a family of so‐called zeotype POM frameworks. The compound class is accessed by electrostatic assembly of metal–oxo cations (e.g. [Cr_3_O(OOCH)_6_(H_2_O)_3_]^+^ (=**{Cr_3_}**)) with Keggin‐type POM anions (e.g. [SiW_12_O_40_]^4−^ (=**{SiW_12_}**)) and stabilizing alkali/alkaline earth metal cations.^77^ The open‐pore frameworks showed intriguing size‐selective uptake of small molecules as well as proton conductivity and heterogeneous oxidation catalytic activity.^78^ Interestingly, the interactions between the metal–oxo cations and POM anions are mediated by the presence of alkali/alkali earth metal cations. This leads to a complex structural interplay between the three components as illustrated for the prototype compound K_3_[Cr_3_O(OOCH)_6_(H_2_O)_3_][α‐SiW_12_O_40_]⋅16 H_2_O.^77^ Here, the K^+^ cations form stabilizing linkages between the **{Cr_3_}** cations and the **{SiW_12_}** anions, facilitated by the *C*_3_‐symmetry matching of the cationic and anionic components (Figure 3).

The direct coordinative linkage of POMs via metal cations embedded within the cluster shell has been reported by Cronin and colleagues as a means to access porous, redox‐active 3D POM‐OFs, so‐called Keggin‐nets.^79^ The authors show that incorporation of Mn^III^ ions into lacunary Keggin‐type tungstates leads to trigonal or tetrahedral linkage nodes where three or four Mn−O−W coordination bridges directly link neighboring Keggin anions as shown in Figure 3. This linkage mode is unusual as it results in a dense packing of Keggin anions while at the same time forming a 3D‐porous architecture. The negative charge of the inorganic framework is balanced by organo‐cations within the pores (e.g. morpholinium). In several reports the authors showed that the framework pores are accessible for cation exchange and that the Mn^III^ cations within the framework can be completely and reversibly reduced to Mn^II^. In addition, other 3d transition metals, for example, Co ions can also be used as linkages, leading to isostructural frameworks with different reactivity.^76^

### 2.3 POM Capsules as Hosts for Cationic Guests

In addition to linking POMs into larger, possibly porous frameworks, POMs themselves can act as anionic, porous architectures. Seminal studies by Müller and others have examined the molybdenum‐based Keplerate “spheres”^80^, ^81^ (e.g. [[Mo_72_^VI^Mo_60_^V^O_372_(CH_3_COO)_30_(H_2_O)_72_]^42−^=**{Mo_132_}**) with respect to their supramolecular reactivity. The authors examined the 20 {Mo_9_O_9_} pores (diameter ≈0.35 nm) on the surface of **{Mo_132_}** (Figure 3) and showed that they enable the uptake and release of cationic (e.g. ammonium, metal ions), anionic (e.g. carboxylate) and neutral (e.g. water) species.^81^ Of particular interest is the selective blocking of the *C*_3_‐symmetric pore windows, for example, by guanidinium. These cations combine electrostatic and hydrogen‐bonding interactions with matching *C*_3_‐symmetry and complementary size to fit the metal–oxide pores in **{Mo_132_}** (Figure 3). Based on this guest exchange behavior of **{Mo_132_}**, the authors describe them as artificial inorganic cells to explore biologically relevant ion‐gating and transport.^81^

Other POM structures have been considered as pore models to examine the uptake and transport of cations. Kortz and co‐workers have explored the metal cation binding in the crown‐shaped [P_8_W_48_O_184_]^40−^ (=**{P_8_W_48_}**) and demonstrated that up to 20 Cu^II^ ions as well as a wide range of other metal cations can be encapsulated at precise positions within the central pore (diameter ≈1 nm) of this species.^82^ The study laid the foundation for the use of **{P_8_W_48_}** as building block for porous frameworks: Cronin and co‐workers linked **{P_8_W_48_}** with metal cations (coordinated to external positions), producing a family of 3D porous frameworks with accessible pores suitable for cation uptake and release.^83^, ^84^ These framework materials could conceptually bridge modular‐assembled frameworks such as metal–organic frameworks and inorganic porous materials such as zeolites. The combination of inorganic metal‐oxo building units and controllable structure assembly could lead to new reactivity and applications.

### 2.4 Cation‐Controlled POM Surface‐Deposition and Growth

The cation‐induced linkage of POMs into stable lattices is not only important for POM‐OF framework design, but holds great promise for integrating solid‐state POMs into devices for advanced technologies. This approach has recently received significant interest for the electrical “wiring” of POMs to electrodes to form electroactive composites for electrochemical energy conversion and storage.^3^ In an instructive example, Song, Streb and co‐workers hydrothermally deposited Co^2+^/Ni^2+^‐linked Dexter–Silverton tungstates ([Co_6.8_Ni_1.2_W_12_O_42_(OH)_4_(H_2_O)_8_]) as microcrystals on metallic nickel foam electrodes.^85^ In the crystal lattice, the M^2+^ cations (M=Co/Ni) adopt two different linkage modes: one cation position links two adjacent tungstate anions by coordination to three terminal W=O units on each cluster, giving an octahedral environment around M^2+^. The second cation position also links two neighboring clusters. Here, each cluster coordinates to the metal cation via μ^2^‐bridging oxo ligands located on opposite apical positions of the coordination octahedron. The four equatorial positions are occupied by aqua ligands. The resulting composite electrocatalysts showed high reactivity and stability for alkaline water oxidation (pH≈13). The Co^2+^/Ni^2+^ ions provide multi‐electron redox‐activity required for the proton‐coupled water oxidation. In addition, they provide a stable 3d‐linked crystal lattice which renders the material insoluble, even under the harsh alkaline conditions employed.

An alternative immobilization approach was reported by Bonchio, Prato and colleagues, who used a covalent electrode‐surface functionalization route to develop POM composite water oxidation electrocatalysts:^86^ the authors covalently functionalized conductive multi‐walled carbon nanotubes with polycationic polyamidoamine (PAMAM) surface groups and used these to electrostatically anchor anionic POM water oxidation catalysts ([Ru_4_(H_2_O)_4_O_4_(OH)_2_(γ‐SiW_10_O_36_)_2_]^10−^. The composite material (when deposited on indium tin oxide electrodes) showed sustained water oxidation to molecular oxygen at neutral pH in phosphate buffer. The performance of the catalyst (based on turnover frequency) was only slightly lower compared with the system under homogeneous conditions, highlighting that stable electrostatic immobilization of POMs on nanostructured carbon electrodes is possible with retention of the catalytic activity. While PAMAM cationic groups negatively affect electronic conductivity, subsequent studies showed that non‐covalent association of counter‐cations (e.g. pyrene‐functionalized ammonium ions) by π‐stacking is also possible.^87^

## 3 Supramolecular POM‐Cation Aggregation Leading to Soft Matter

In this section, we discuss the supramolecular assembly of POMs with counter‐cations that promote unusual nanoscopic, microscopic or macroscopic structure, sometimes leading to function. With the exception of the metal‐cation promoted blackberry formation, the studies described are mostly focused on quaternary alkyl ammoniums which are widely used as organic counter‐ions for POMs.

### 3.1 Metal‐Cation Promoted Aggregation: Blackberry Structures

**Figure 4:**
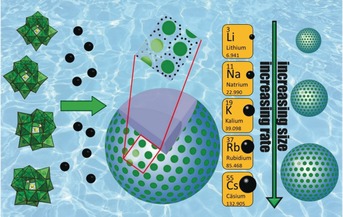
Schematic of “blackberry” macromolecular self‐assembly. From left to right: dilute solutions of POMs (green polyhedral representation of the Keggin ion) with added alkali metal cations (black spheres) enable assembly of the blackberry structures (hollow capsule; green dots: POMs, see inset for close‐up view of the POM environment) Right: the size of the blackberries and the assembly rate increase with increasing alkali metal cation radius. (Blackberry cartoons courtesy of Tianbo Liu).

The so‐called blackberry structures were discovered by Tianbo Liu in the early 2000s,^88^ employing the large molybdate wheel polyanion **{Mo_154_}** (=[Mo^VI^_126_Mo^V^_28_O_462_H_14_(H_2_O)_70_]^14−^). Blackberry structures, now well‐known amongst the metal–oxo cluster community, are solution‐phase hollow capsules containing hundreds to thousands of POMs that are associated by counter‐cations in a single curved layer, comprising the blackberry surface (Figure 4). Since their initial discovery, Blackberry formation is now accepted as a common phenomenon for inorganic POMs^89^, ^90^ and for amphiphilic POMs that are functionalized by hydrophobic organic “tails”.^91^, ^92^, ^93^, ^94^, ^95^ Also noteworthy, the uranyl peroxide polyoxometalates of several distinct geometries and compositions,^52^, ^96^, ^97^, ^98^ as well as the noble metal POMs,^99^, ^100^ also assemble into blackberry structures, indicating the universality of the underlying structure formation processes. Beyond the scope of this Review, blackberry structures have also assembled from cationic coordination compound nanocages.^101^

Blackberries are generally 10 to 1000 nm in diameter, and it is presumed that the POMs generally organize on the capsule surface in hexagonal‐packed arrays, not unlike the drupelets of blackberries. However, only short‐range order has been shown by TEM imaging,^88^, ^102^ while the introduction of curvature might require regions with different packing motifs. Important to this Review, residing between the POMs on the blackberry surface there must be counter‐cations, otherwise the repulsive electrostatic interactions between neighboring POMs would overwhelm any attractive forces. Blackberry formation only occurs in dilute solutions ([POM] ≈0.1–10′s of mg mL^−1^), and their formation can be triggered by time, addition of a less polar solvent, or addition of counter‐cations with low hydration energy (Rb^+^, Cs^+^) or high charge (Mg^2+^/Ca^2+^/Sr^2+^/Ba^2+^,^103^ Al^3+^, Y^3+^). These cations are effective because they can undergo strong contact ion‐pairing with POMs in solution (see Section 1.3), which is the first step towards blackberry formation. The size of the blackberries that form is also dependent on the type of counter‐cation; larger alkali metals or divalent cations with strong association trigger formation of larger assemblies. One explanation for this is rooted in the anion–anion repulsion that occurs as POMs approach each other on a highly curved surface. By retaining less curvature (and larger macrostructures), attractive interactions within the curved shell become dominant and facilitate large blackberry formation.^49^, ^104^ Not surprising, counter‐cations can and do also enter the water‐filled interior of the blackberries.^105^ In the original study of **{Mo_154_}** blackberries, decreasing pH increased the size of the blackberries, indicating H^+^ can also serve as the intermediary in this self‐assembly process.

Unlike tungstate and molybdate POMs, several uranyl peroxide capsules,^52^, ^96^ self‐assemble in base rather than acid, and the capsules exhibit no acid–base chemistry, further solidifying the importance of the metal‐counter‐cations, usually alkali metals. Since the uranyl peroxide POMs possess a hollow‐capsule topology, alkali metal (and other) counter‐cations can exchange from the capsule inside into solution and vice versa.^106^, ^107^ Without the encapsulated cations, the charge of the uranyl peroxide POMs is quite high (generally equal to the number of uranyl ions; that is, **{U_60_}** ^60−^ (=[(UO_2_(O_2_)OH)_60_]^60−^), which does not allow blackberry formation since hydrophobic interactions are important for this self‐assembly. Therefore, adding counter‐cations to these solutions to trigger blackberry formation both drives the alkali metal cations into the encapsulated state for neutralization, and bridges the anions in the blackberry structure. Finally, blackberry formation can be influenced by the interplay between acid–base chemistry and alkali metal cations. Blackberry assembly of iron molybdate and chromium molybdate POMs was promoted by NaOH addition, which deprotonated acidic water bound to the Cr or Fe.^108^ In this case, the Fe^3+^–Mo POMs assemble more rapidly since Fe^3+^ is more acidic than Cr^3+^. Meanwhile the added Na^+^ resides between the POMs on the blackberry surface, displacing the ammonium cations of the original POM salt.

### 3.2 Quaternary Ammonium Promoted Aggregation: Ordered and Disordered

There are numerous quaternary alkyl ammoniums or quaternary alkyl phosphoniums (QAAs and QAPs respectively, abbreviated QA(AP)s herein when referring to both) readily purchased from commercial suppliers. These include alkyl and aryl derivatives, surfactants, amino acids, poly‐ammoniums (containing more than one ammonium in a molecule), and dendrimers. POMs plus QA(AP)s lead to nearly infinite derivative compounds, and the remainder of this section discusses supramolecular assembly of these. QAAs first utilized in POM chemistry are simple tetraalkyl ammoniums including tetramethyl (TMA), tetrabutyl (TBA) and tetrahexyl (THA) ammonium that can modulate solubility of POMs. TMA is a notoriously “innocent” counterion that does not promote aggregation and allows high solubility and stability in water, particularly for polyoxoniobates and tantalates.^30^, ^109^, ^110^, ^111^, ^112^ On the other hand, TBA and THA have been commonly used for decades to extract (or precipitate) POMs into nonaqueous solvents for numerous purposes including electrochemical studies and applications involving POM redox chemistry,^3^ catalytic studies in organic solvents,^113^ and to stabilize water‐ or pH‐sensitive POMs.^114^ While TMA salts of POMs generally crystallize well for structure elucidation, the longer‐chain alkylammonium salts are notoriously difficult to crystallize, due to the flexibility and lack of order of the alkyl chains, and the weak intermolecular interactions in the solid‐state. These physical characteristics are the basis for many of the complex QA(AP)‐POM phases discussed below.

#### 3.2.1 POM Ionic Liquids

POM ionic liquids (or POM‐ILs) are simply POMs with counter‐cations that afford a melting temperature below 100 °C.^24^ The preparation of these is extremely facile: the POM‐ILs can typically be obtained by extraction of an aqueous solution of the desired POM with an organic (often toluene) solution of the QA(AP)‐cation of choice. Dietz and Antonio and co‐workers designed POM‐ILs from simple polyoxotungstates; phosphotungstic acid [PW_12_O_40_]^3−^ and the Lindqvist ion [W_6_O_19_]^2−^, with the targeted application of electrochemical devices including fuel cells, capacitors and batteries.^115^, ^116^ They found the QAP derivatives have lower melting temperatures (as low as −48 °C), higher thermal stability, suitable conductivity and reversible electrochemical response. POM‐ILs have found use in catalytic reactions in organic solvents, but the POM does not always serve as the active catalyst. For example, phosphotungstic acid combined with sulfonic acid‐functionalized QAAs catalyze esterification reactions.^117^ The functionalized QAA provides the acid for this reaction. The attractive feature of this POM‐IL is that it is soluble at the beginning of the catalytic cycle, but phase‐separates as the ester concentration increases in solution, providing the benefits of both homogeneous and heterogeneous catalyst. The same materials plus the [PMo_12_O_40_]^3−^ analogues were used for desulfurization reactions;^117^ but it is not clear if either the anion or cation of the POM‐IL plays any role in the catalytic reaction, other than binding the peroxide that is the oxidant.

More recently, Streb and co‐workers have prepared POM‐ILs from lacunary Keggin derivatives including [SiW_11_O_39_]^8−^ and [α‐PW_9_O_34_]^9−^ and simple QAAs, predominantly tetrahexyl‐, tetraheptyl‐, tetraoctyl‐, and trihexyl(tetradecyl)‐ammonium cations.^25^, ^118^, ^119^, ^120^ Their targeted applications included water filtration and treatment,^118^, ^120^ and anticorrosive/antibacterial coatings for metals and building stone.^25^, ^119^ In these applications, both components of the POM‐IL were engaged in the application. Lacunary POMs which feature accessible metal cation binding sites, served to remove heavy metal contaminants in water treatment, while the organic cation attracts organic contaminants by hydrophobic interactions. The medium‐chain alkyls of the QAA can also penetrate and destroy cell membranes (biocidal function) in both applications, particularly as bacteria can be one of the greatest aggravators in degradation of building stone. The POM‐IL coatings also protected stone against acid‐rain, and the coatings proved acid‐stable. This characteristic is most likely due to the good surface‐adherence and water‐repellency of these “tacky” materials.

**Figure 5:**
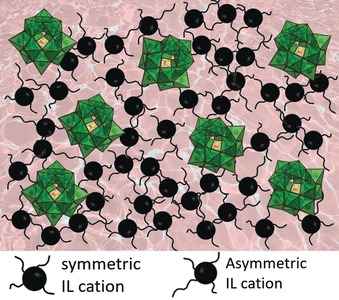
Illustration of a model POM‐IL with a) symmetric QAAs (black), using the Keggin ion (green polyhedra) as a generic POM model. Little is known about the structure and interactions between the POMs and the QAAs in the POM‐IL liquids, this represents opportunity for fundamental discovery and optimization of these compounds. This cartoon depicts a presumed lack of order in the liquid, with POM–POM, QAA—QAA, and POM–QAA interactions.

Distinctly missing from the relatively modest POM‐IL literature are structure/composition‐function relationships, and this represents an opportunity for both rich fundamental research and ability to tailor formulation to function. We expect that important macroscopic materials properties including rheology and melting temperature are controlled by the interactions (or lack thereof) in the liquid state, including POM–QA(AP); POM–POM and QA(AP)–QA(AP) (Figure 5). These putative interactions are in turn highly sensitive to the size, chemical structure, and charge of the cation and the anion, as well as the cation:anion ratio. Even more complex systems featuring several different cations or anions can be envisaged. Considering these factors, it is obvious that the huge library of potential POM‐ILs could benefit from predictive computational and machine learning studies.

#### 3.2.2 Surfactant‐Encapsulated POMs and POM–Surfactant Arrays

The field of POM–surfactant hybrids is closely linked to POM‐ILs and often uses cationic organo‐ammonium surfactants featuring one or two long hydrocarbon chains (generally C_12_–C_20_). The most commonly employed surfactants include DODA (double‐chain dioctadecyldimethyl ammonium) and CTA (single‐chain cetyltrimethyl ammonium, cetyl=hexadecyl). Generally the bulky DODA chains lead to soluble surfactant encapsulated clusters (SECs) that can be deposited as Langmuir films.^121^, ^122^, ^123^ On the other hand the CTA surfactant has led to single‐crystalline structures^124^, ^125^ that provide insight into the arrangement of POMs and QA(AP)s in numerous hybrid phases discussed in this section. The SECs with their bulky shell of long‐chain hydrocarbons can be dissolved in organic solvents and these can be transferred to a surface for molecular devices with electrochemical or electrochromic response, catalytic function,^123^, ^126^ gas adsorption,^123^ or Li^+[127]^ or H^+[128]^ conduction for energy conversion and storage. Organically‐soluble SECs have also been proposed for applications including stabilization of emulsions,^129^ extraction and separation of uranium^130^ and NMR paramagnetic relaxation reagents.^131^

**Figure 6:**
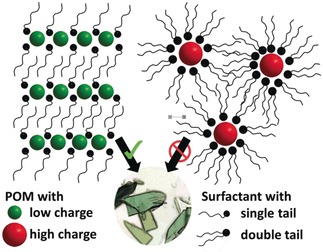
Illustration the different assemblies of POMs plus surfactant QAAs. Surfactants can be either single‐tail (left) or double‐tail (right). Left: The POMs that are small with low charge (i.e. −2 to −4; green spheres) can readily form lipid bilayers where the main forces of the assembly are strong interdigitation of the parallel surfactant tails, and also electrostatic attraction between the POM anions and the small ammonium cation heads. These lipid bilayer assemblies are conducive to crystal formation (middle). Right: Larger POMs with higher charge (red spheres) form surfactant‐encapsulated clusters (SECs; shown with double‐tail surfactants). These assemblies do not have strong interactions between the lipid tails, due to curvature, rather than parallel orientation; and the lack of order and strong interactions prohibits crystallization.

In the spirit of this comprehensive Review about POM‐counter‐cations, we will not go in depth into SEC applications, as this has been recently reviewed.^132^ Instead, we will discuss what is known about the structures of both ordered (crystalline) and non‐ordered cluster‐surfactant phases derived from POM‐counter‐cation aggregation (see Figure 6), and how this can lead to tailoring these hybrid materials for applications such as ion conductivity or energy storage. The structure of SECs has been inferred by SAXS in solution, as well as X‐ray reflectivity, TEM and molecular modeling of Langmuir layers.^130^, ^133^ For surface‐deposited Langmuir layers, cluster‐surfactant phases, single‐crystal cluster‐surfactant phases, and SECs in solution, we can generally conclude that the size of the POM controls the resulting organization of POMs and surfactants in the solid‐phase. In the solid‐state, the cationic ammonium heads of the surfactant electrostatically aggregate around the anionic POM, in essence forming an inverse micelle where the POM anion is the hydrophilic core, and the long alkyl chains extend away from the POM, forming the hydrophobic exterior. For large POMs (diameter≈≥2 nm with higher charge), the interaction between the SECs via surfactant tail interdigitation is weak. This is because in this arrangement, the tails extend radially from the central POM and are not aligned in a parallel fashion. Weak interactions between SECs leads to higher solubility in organic phases, poor crystallization behavior, and hexagonal close packing (hcp) within Langmuir layers. Smaller POMs (diameter≈≤1 nm with lower charge), promote crystallization of lamellar layers. In these phases, bilayers of strongly interdigitated surfactants alternate with layers of hexagonally close‐packed POMs (Figure 6). Again the interaction between the POMs and surfactant ammonium heads is electrostatic; and because the POMs are small, the hydrophobic interactions between well‐ordered, parallel surfactant tails is strong.

Nyman and co‐workers^124^ noted that the ratio of one POM cluster per four surfactant chains (i.e. two DODAs or four CTAs per POM) promoted crystallization most readily. This was further confirmed in attempts to crystallize two‐electron reduced silicomolybdate: [SiMo_12_O_40_]^6−^ with CTA: The diprotonated form, [H_2_SiMo_12_O_40_]^4−^ crystallized instead. Similar examples have been shown including crystallization of [H_2_V_10_O_28_]^4−^ with a C_10_ surfactant,^134^ and [Li_2_V_10_O_28_]^4−^ with a C_8_ surfactant.^127^ These phases all represent crystal engineering opportunities to obtain POM‐based materials for proton conduction, lithium conduction, or simultaneous ion–electron conduction for battery electrodes.

#### 3.2.3 Other POM‐QA(AP)s

We will conclude this discussion of POM‐QA(AP)s with a few miscellaneous examples of secondary supramolecular assembly. Dendrimers are multiply branched polyammonium cations featuring a number of cationic ammonium sites for electrostatic binding and hydrogen bonding of POMs. Charge‐neutral dendrimer–POM assemblies of one, two, three, and four POMs per dendrimer have been assembled from mono‐, bi‐, tri‐, and tetra‐directional dendrimers.^135^ These architectures have been exploited as catalysts that are readily recovered from organic media, providing the advantages of both heterogeneous and homogeneous reaction operation. Neumann and co‐workers for example^135^ used the peroxophosphotungstate [PO_4_{WO(O_2_)_2_}_4_]^3−^ linked with dendrimers to act as catalyst for the epoxidation of cyclooctene. They noted that the catalytic behavior was similar for all of the POM‐dendrimers; but those with three or four POMs were recovered more readily from solution by precipitation using a poorer solvent. Gröhn and co‐workers^136^ created coiled dendrimer–POM assemblies (determined from light and neutron scattering of aqueous solutions) from a generation‐4 poly(amidoamine) (PAMAM) dendrimer and phosphotungstic acid. Aqueous solubility was achieved by titrating the dendrimer with sub‐stoichiometric POM concentrations so that the assembly retained an excess positive charge. These assemblies exhibited photoactive behavior as demonstrated by the photooxidative degradation of common organic dyes. Notably, the aggregates showed significantly higher photooxidative activity compared to the pure POM, which the authors associated with different binding and aggregation behavior of the dyes in the presence of POM‐dendrimer species. Although POM‐dendrimers represent an opportunity for beautiful supramolecular assembly (as exemplified by the Gröhn study^136^) and derivative applications, further reports have been relatively sparse thus far. Contributing factors could be the expense of purchasing, or difficulty in synthesizing dendrimers together with challenges in preventing POM‐dendrimer “over‐aggregation” and subsequent precipitation.

In the laboratory of Wang,^137^, ^138^ Wells–Dawson POMs and functionalized derivatives ([P_2_W_18_O_62_]^6−^ and [Pd_2_(P_2_W_17_O_61_)_2_]^8−^) combined with both TBA and CTA yield “nanorolls” as shown by TEM. Characterization by NMR spectroscopy showed the TBA and CTA to be present in these assemblies in a 1:1 ratio. These supramolecular assemblies are intermediates between the lamellar layers of POM‐surfactant crystals of smaller POMs, and the SECs of larger POMs. The authors suggest that the assembly of curved layers drives the nanoroll formation. The assemblies were also post‐functionalized by polymerization to produce a rigid material^138^ and can be used for catalysis.^137^ The study suggests that cations with different size and shape could be combined with POMs to develop novel structure and function beyond the classic POM–surfactant systems known to‐date. In addition, careful control of the number and length of the cation hydrophobic “tails” could be a simple tool to manipulate cation‐tail interdigitation and thus the resulting supramolecular assembly. In the present example, the bulky, spherical TBA counter‐cations may force radiating orientation of the CTA surfactant tails, thus preventing parallel alignment that is characteristic of the layered POM–surfactant crystals (Figure 3).

Cronin and colleagues have shown that POM crystal dissolution in the presence of polyaromatic cations can be used to access tubular microstructures.^139^, ^140^ The tube self‐assembly proceeds via a membrane mechanism similar to the well‐known “chemical garden”. A semi‐permeable membrane is formed by POM‐polyaromatic cation precipitation, and osmotic pressure‐controlled solvent diffusion through the membrane leads to membrane rupture and further tube growth. The direction of growth can be controlled by external stimuli such as local heating and convective currents in the solution. A later study generalized the concept by using various organic cations and POM anions. ^[141]^

### 3.3 Cation‐Induced Formation of New Cluster Structures

As described in Section 1.4, there is no doubt that alkali metal counter‐cations template formation of POM architectures by stabilizing lacunary holes. Higher‐valent metal cations also serve this role in allowing isolation of unusual POM geometries or chemistries. However, examples with organic ammonium cations are far fewer: in one striking example, Cronin and colleagues^142^ isolated an unprecedented, asymmetric Mo‐POM, [H_2_Mo_16_O_52_]^10−^, by using the adamantane‐shaped protonated hexamethylene tetramine. A space‐filling representation shows that the cluster is “shrink‐wrapped” in the organic counterions. To the best of our knowledge, this cluster has not been obtained with any other counter‐cation. Other proclaimed examples of organic counter‐cations that isolate new POM geometries instead exhibit related but different phenomena including organic ligation of new POM geometries, unusual linking motifs of known POM fragments, or assembly of known POMs into framework materials. The few examples of cation‐mediated formation of new POM architectures represents an outstanding research opportunity, particularly when targeting novel iso‐POMs, for example, for Group V metals.

## 4 Emerging Areas and Perspectives

The previous sections have highlighted that the role of cations in POM chemistry goes beyond simple electrostatic charge‐balancing. In this final section, we will provide a brief outlook of emerging directions, where functional cations interact with POM anions, leading to unexpected properties and reactivities.

### 4.1 Cation‐Controlled POM Assembly and Reactivity

**Figure 7:**
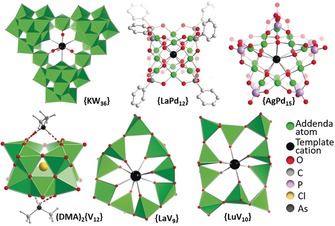
Structural representation of the cation‐templated POMs described in Section 4.1.

Traditionally, POMs assemble around anion templates including halides and oxoanions. However, numerous examples have emerged in which POM anions are templated by internal metal cations. In a combined experimental and theoretical study, Kortz, Poblet, and colleagues described the templating of polyoxopalladates by M^3+^ cations (M=La^3+^, Ga^3+^, In^3+^; Figure 7).^143^ Their analyses show that the size and charge of the metal cation, the dehydration energy, and the electrostatic interactions between cation and polyoxopalladate fragments control cluster assembly. This level of insight is critical, as it provides clear control parameters for the targeted development of fundamentally new cluster architectures.

Also using lanthanides as internal templates, Hayashi and colleagues have reported a series of POMs where ring‐shaped vanadates based on corner‐sharing [VO_4_] tetrahedra are assembled around a central lanthanide(III) cation.^144^ The authors beautifully demonstrate that the ionic radius of the lanthanide cation modulates the cluster architecture by its preferred coordination geometry (Figure 7). For example, the larger 8‐coordinate lanthanum cation (radius=1.16 Å) yields a **{LaV_9_}** species, whereas the smaller 6‐coordinate lutetium cation (radius=0.86 Å) templates **{LuV_10_}**. Initially this seems counterintuitive. However, in **{LaV_9_}**, only one [VO_4_] tetrahedron is not coordinated to the lanthanum cation, while **{LuV_10_}** features four peripheral [VO_4_] units not linked to the central template, leading to a larger, less rigid structure (Figure 7). The work also highlights that while prediction of the cation coordination environment seems possible, prediction of the resulting cluster architecture and structure is more challenging. Notably, for these systems, it is thus far not clear whether the templating process occurs through the (solvated) cationic metal species, or whether neutral or even anionic intermediates (e.g. hydroxo or oxo complexes) could be formed under the given reaction conditions.

Streb and colleagues recently reported the synthesis of dodecavanadate anions featuring two metal binding sites blocked by NH_2_Me_2_^+^ placeholder cations: (NH_2_Me_2_)_2_[V_12_O_32_Cl]^3−^ (**(DMA)_2_{V_12_}**, Figure 7). Intriguingly, the species is only formed when In^3+^ or Al^3+^ are present in the reaction solution; however, in the crystalline product, no traces of these metals are observed crystallographically or by elemental analyses.^145^ This suggests that these high charge‐density, Lewis acidic cations might play a templating or structure‐directing role, possibly enabling the aggregation of smaller, reactive vanadate fragments^146^ to form the stable dodecavanadate structure. In addition, the presence of dimethyl ammonium cations during cluster assembly is critical to block the vacant metal binding sites. Subsequent exchange of these placeholders with metal cations enables controlled functionalization of the cluster architecture.^145^, ^147^, ^148^, ^149^

A beautiful example of cation‐templated iso‐polyoxotungstate formation was described by Cronin and colleagues who showed that the triangular **{KW_36_}** species (K(H_2_O)_4_[H_12_W_36_O_120_]^11−^, Figure 7) can be formed using a central K^+^ cation.^150^ Cluster formation was only observed in the presence of potassium cations (which were initially leached from the reaction glassware). Notably, the presence of a second cation, triethanol ammonium, was also required for the **{KW_36_}** synthesis and its absence led to the formation of different cluster architectures. While the role of this cation is currently unclear, it is likely a combination of electrostatic, hydrogen bonding (via ‐OH and ‐NH groups) and possibly even coordinative interactions (via ‐OH groups) could stabilize reactive fragments and lead to **{KW_36_}** formation. In later studies, the authors also showed that replacing the K^+^ with larger alkali and alkaline earth metals, and other ammonium or primary organo‐ammonium groups is also possible, opening the door to organo‐functionalized species.^151^

The interplay between pH and counterions normally control POM speciation; but Nyman and co‐workers decoupled these processes and demonstrated that alkali metal cations alone can promote Nb‐POM disassembly and reassembly.^46^ Decaniobate, **{Nb_10_}**, is an unusual POM that has neither acidic nor basic character. Addition of small amounts of alkali metal salts to aqueous **{Nb_10_}**, even in a neutral buffer solution, leads to disassembly of **{Nb_10_}** into **{Nb_7_}**, which reassembles into **{Nb_24_}** species. Further illustrating the role of the alkali metal counterion, the rate of disassembly–reassembly increases down the alkali metal series. It is likely alkali metals promote reactivity and speciation of other POM solutions, but the exact nature of their role is difficult to isolate in these complex and dynamic solutions.

### 4.2 Cation‐Directed POM Superstructures

The aggregation of individual POM anions into larger superstructures holds great promise for the bottom‐up development of nanostructured functional materials, particularly as this approach can give access to systems in the 1–10 nm size range which is difficult to access by other methods.^152^ An intriguing example was recently reported by Izzet and colleagues; the authors used a Dawson‐POM covalently functionalized with two terpyridine metal coordination sites, giving a cluster with approximate Y‐shape.^153^ The authors introduced iron(III) counter‐cations which coordinate to the terpyridine units and in DMSO solvent trigger the assembly of discrete triangular supramolecules with a diameter of 5 nm. Structural assignment was based on NMR spectroscopy, small angle X‐ray scattering (SAXS) and theoretical calculations. Upon addition of less polar solvents (MeCN, acetone) to the triangle solution, assembly of well‐defined spherical nanoparticles containing about 18 triangular units is observed. The authors attribute this to an electrostatic aggregation between positively charged (Fe^3+^‐based) and negatively charged (POM‐based) regions of the triangles. While the individual molecules are stabilized by the highly coordinating solvent DMSO, addition of less stabilizing solvents leads to nanoparticle formation. The process is reversible and was monitored by NMR spectroscopy, TEM, and SAXS.

The challenges arising from POM–cation combinations in functional systems have been demonstrated by Streb, Rau, and colleagues who explored the solution aggregation and colloid formation between redox‐active POM anions (in the context of water oxidation catalysis) and various cationic metal complex photosensitizers bearing aromatic ligands (e.g. [Ru(bpy)_3_]^2+^, bpy=2,2′‐bipyridine).^50^ In most cases studied, the authors observed ion pairing and electrostatic colloid formation between the ionic species under conditions typically used in water oxidation catalysis. In addition, colloid formation is strongly dependent on the ionic strength of the solution, and colloid dissolution (and regeneration of the homogeneous molecular distribution) was achieved by simple addition of inorganic salts such as NaCl. The study highlights that technologically important processes can be significantly affected by POM–cation interactions in solution so that detailed analyses of these complex systems are required when performing reactivity studies.

### 4.3 POM–Biomolecule Composites

POM interactions with cationic regions of biomolecules such as peptides, proteins, or ribosomes have been used for crystallographic structure elucidation and played an important role in the 2009 Nobel Prize for Chemistry.^154^, ^155^ While this behavior led to groundbreaking insights in structural biology and biochemistry, the principles have not been extensively used for the design of functional bioactive hybrid materials. Over recent years, pioneering work has showcased how POMs can be integrated as structural and reactive components in bio‐inorganic hybrids, where electrostatic interactions between POMs and cationic moieties of the biomolecules are key to self‐assembly processes.

**Figure 8:**
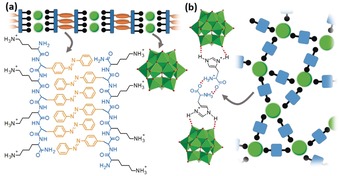
a) Nanofiber formation by electrostatic aggregation between [SiW_12_O_40_]^4−^ and cationic oligopeptides capable of π stacking.^156^ b) Adhesive formation by electrostatic and hydrogen‐bonding aggregation between [SiW_12_O_40_]^4−^ and hydrogen‐bonded cationic histidine dimers.^157^

In a recent study, Lee and colleagues developed a new approach to nanostructured antibiotic organic–inorganic hybrids: oligopeptides featuring cationic ammonium sites for charge‐balance as well as aromatic (e.g. phenyl) side‐chains capable of π‐stacking were combined with Keggin polyoxotungstates.^156^ The combination of electrostatic aggregation (between POM and ammonium groups) as well as π‐stacking (between aromatic sidechains) led to the self‐assembly of high aspect‐ratio nanofibers with widths of ≈5 nm and lengths of several hundred nm (Figure 8). The nanofibers showed antimicrobial action against *E. coli* bacteria and mechanistic studies suggest that the fibers cause cell membrane damage, leading to cell death. Remarkably, the nanofibers also show significantly higher stability against enzymatic degradation compared to the oligopeptide precursors, so that novel approaches to antimicrobial agents could become possible.

Using related assembly principles between POMs and biomolecules, Li and colleagues recently mimicked the wet‐adhesives produced by certain sessile organisms (e.g. mussels)^157^ by combining the Keggin POM [SiW_12_O_40_]^4−^ with the cationic amino acid histidine as counter‐cations. Crucially, the assembly of both components was carried out in aqueous media at pH 3, where the imidazole sidechain of histidine is protonated and the α‐amino acid remains in its zwitterionic state, leading to an overall +1 charge and a POM:histidine ratio of 1:4. Aggregation was therefore driven by electrostatic aggregation between the POM and the imidazolium ring of the histidine and also by intermolecular hydrogen‐bonding between the amino acid zwitterions (Figure 8), so that the combined action of both linkage modes gives a material with excellent adhesive properties on various substrates, even when wet. In addition, the adhesive showed reversible electrochromic properties (due to reduction of the POM) so that functional materials with multiple properties become accessible. The modular design approach allows independent tuning of cation and anion properties, so that applications in bio‐medicine, surface modification or even tissue engineering can be envisaged. In future, this approach could be taken one step further still, as POMs have recently been incorporated covalently into oligopeptide chains by solution and solid‐phase syntheses.^158^ This could be used to design POM–peptide aggregates where molecular function such as redox‐ or catalytic activity is combined with supramolecular recognition (POM–peptide or peptide–peptide). Consequently, novel structure and function could be accessed from the molecular through to the micrometer scale.

### 4.4 Heterogeneous Cationic Matrices

Using cationic heterogeneous substrates for the electrostatic immobilization of POMs can be an excellent tool to access technologically important composites.^159^ This approach is particularly important when the cationic substrate and the POM anion introduce synergistic reactivities which lead to multi‐functional composites. A prime example of this material combination was recently reported by Li, Zang and colleagues. The authors used a cationic covalent organic framework (COF) based on cross‐linked ethidium bromide units.^160^ The compound features accessible cylindrical pores (diameter ≈1.5–1.8 nm) which contain halide ions to balance the cationic charge of the COF. Exchange of the halide anions with Keggin polyoxotungstates led to a ≈100‐fold increase in proton conductivity at room temperature. Additionally the composite exhibits high chemical and thermal stability and increased water retention, useful for proton conduction membranes in fuel cells. In a related study, Li and colleagues showed that POM anions can be used to modulate the structure of cationic block copolymer matrices.^161^ While the non‐functionalized block copolymer (PS‐*b*‐P2VP, poly(styrene‐*block*‐2‐vinylpyridine)) forms well‐behaved lamellar structures, POM incorporation leads to the formation of bicontinuous nanocomposite structures with phase separation on the <100 nm scale. The authors suggest that the POM ([SiW_12_O_40_]^4−^) promotes micellar structures within the block copolymer formed by protonation of the pyridine groups and subsequent electrostatic aggregation between POM and the resulting pyridinium cations. The nanostructured composite showed high proton conductivity over a wide temperature range, as well as higher mechanical and thermal stability compared with the non‐modified block‐copolymer. This approach therefore offers new material design concepts, where molecular species are used to control structure and function of nanoscale composites.

A conceptually related approach has been developed for layered double hydroxides (LDHs) which have been explored as heterogeneous supports for reactive POMs. LDHs are 2D cationic metal hydroxide layers formally derived from the mineral brucite (Mg(OH)_2_), where cation replacement (e.g. Al^3+^ for Mg^2+^) or cation oxidation (e.g. Fe^2+/3+^) leads to a net cationic charge within the layer. Charge balance is achieved by intercalation of mobile anions and water in the inter‐layer region. As this region is chemically accessible, ion‐exchange of the original anions with POMs is possible and has been used to access a range of industrially relevant catalysts;^162^ and complex catalytic cascade reactions have also been demonstrated.^163^ In future, the materials design concept could be expanded for potential applications in multifunctional (electro‐)catalysis and battery electrodes.

### 4.5 Polyoxocations and No Cations

Two further research areas related to cations in POM chemistry should be mentioned, which lie somewhat outside the scope of this Review. First, Laskin and co‐workers reported groundbreaking studies on the isolation of POM anions, without cations, using the so‐called ion soft landing technique.^164^ The technology—closely related to electrospray ionization mass spectrometry—enables the deposition of mass‐selected, cation‐free POM anions on solid substrates, such as electrode surfaces. Thus, study of the “naked” anion becomes possible. Pioneering studies have shown that this principle can be used for the bottom‐up construction of model electrodes to rationalize energy storage processes.^165^

Second, pioneering studies have also started to merge the fields of polyoxocation and polyoxometalate chemistry: in early studies, Kwon and colleagues have investigated the electrostatic assembly of aluminum hydroxo cation clusters with POM anions, leading to porous framework materials.^166^ More recently, cationic metal–oxo species, for example, [Bi_6_O_8_]^2+^ or [Pb_8_O_6_]^4+^ have been used as central templates, driving the formation of uranyl polyoxometalate capsules such as [(UO_2_)(O_2_)(OH)]_24_^24−^.^73^ Further, the supramolecular three‐component recognition was studied by combining Wells–Dawson polyoxotungstates, cationic tantalum bromide clusters ([Ta_6_Br_12_(H_2_O)_6_]^2+^) and neutral γ‐cyclodextrin.^167^ In this system, the cyclodextrin acted as central connector between the two oppositely charged cluster units, so that a combination of isotropic electrostatic interactions and directed hydrogen bonding enables the aggregation of complex supramolecular solid‐state structures. A similar electrostatic aggregation of polyoxocations (e.g. [ϵ‐PMo_12_O_36_(OH)_4_La_4_(H_2_O)_10_]^5+^ (**{La_4_Mo_12_}**, see Section 2.1) and classical POMs (e.g. [γ‐SiW_10_O_36_]^8−^) has recently been used for the assembly of membranous tubes and other microstructures which could become relevant for separation or permeation technologies.^168^ Furthermore, it has recently been proposed that structure formation in titanium polyoxocations can be controlled by virtue of the halide counter‐anion employed. This suggests that some of the principles laid out in this Review may be relevant for more extensive development of metal–oxo polycation chemistry.^169^

## 5 Summary and Outlook

Anecdotally, the senior authors of this Review (C.S. and M.N.) have noted in numerous papers and oral presentations the comments of *surprising, unexpected, important*, etc. pertaining to the roles of counter‐cations in POM chemistry, and the chemistry of other metal–oxo clusters. An inspiration for writing this Review was to bring together this body of knowledge in a formal way, to define emerging themes, and inspire future research. While the classic view of POM chemistry describes counter‐cations as alkali metals and alkylammoniums, our advanced definition includes surfactants, biomolecules, polycations, multivalent metal cations, and positively charged surfaces and matrices. Likewise, the classic roles of these counter‐cations are to control POM dissolution, precipitation, and purification. The expanding and emerging roles of POM counter‐cations include templating and stabilization from both the inside and the outside of POMs, framework construction and supramolecular assembly, and promoting reactivity and speciation. We believe these roles have been present and enabled exciting POM chemistry for many decades. However, the recognition and understanding the roles of these counter‐cations are emerging and growing along with advanced instrumentation and computational power. In our view, deliberately recruiting counter‐cation function will enrich and expand fundamental and applied POM science.

## Conflict of interest

The authors declare no conflict of interest.

## Biographical Information

*Archismita Misra received her integrated BSc–MSc dual degree in Chemical Sciences from the Indian Institute of Science Education and Research—Kolkata*, *India in 2015. She then joined the group of Carsten Streb for her PhD studies with a Schlumberger Foundation Faculty for the Future fellowship. Archismita is interested in the development of polyoxometalate ionic liquids (POM‐ILs) for sustainable chemistry applications*.

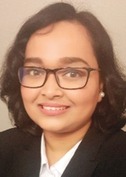

## Biographical Information

*Karoly Kozma received both his BSc and MSc degree at University of Szeged, in Hungary. In 2014, he joined the research group of May Nyman at Oregon State University pursuing his PhD. He is studying aqueous solutions of early transition metals (group IV) and is using combinations of X‐ray scattering methods to elucidate various metal–oxo systems*.

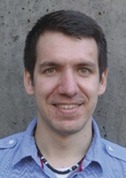

## Biographical Information

*Carsten Streb is Professor of Inorganic Chemistry at Ulm University and group leader at the Helmholtz Institute Ulm. His love for POMs started during his PhD, working with Lee Cronin at the University of Glasgow. His current research is focused on designing POM‐based functional materials and composites to address global chemical challenges including energy conversion/storage, water purification, and public health*.

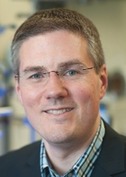

## Biographical Information

*May Nyman is a professor of Chemistry at Oregon State University. She gained her PhD at the University of New Mexico with Prof. Mark Hampden‐Smith. Solid‐state and solution X‐ray characterization of new cluster topologies make May particularly happy. She loves metal–oxo clusters from across the periodic table with a partiality towards Group IV, Nb, and actinides. In addition to understanding fundamental aqueous speciation of metal cations, May's research for 20 years has spanned applications in nuclear waste management, inorganic lithography, and environmental remediation*.